# Diagnosis, Clinical Features, and Self-Reported Morbidity of *Strongyloides stercoralis* and Hookworm Infection in a Co-Endemic Setting

**DOI:** 10.1371/journal.pntd.0001292

**Published:** 2011-08-23

**Authors:** Sören L. Becker, Benjamin Sieto, Kigbafori D. Silué, Lucas Adjossan, Siaka Koné, Christoph Hatz, Winfried V. Kern, Eliézer K. N'Goran, Jürg Utzinger

**Affiliations:** 1 Department of Epidemiology and Public Health, Swiss Tropical and Public Health Institute, Basel, Switzerland; 2 University of Basel, Basel, Switzerland; 3 Center for Infectious Diseases and Travel Medicine, Department of Medicine, University Hospital Freiburg, Freiburg im Breisgau, Germany; 4 Dispensaire Rural de Léléblé, Léléblé, Côte d'Ivoire; 5 Laboratoire de Zoologie et Biologie Animale, Unité de Formation et de Recherche (UFR) Biosciences, Université de Cocody, Abidjan, Côte d'Ivoire; 6 Département Environnement et Santé, Centre Suisse de Recherches Scientifiques en Côte d'Ivoire, Abidjan, Côte d'Ivoire; 7 Hôpital General de Taabo-Cité, Taabo-Cité, Côte d'Ivoire; 8 Department of Medical Services and Diagnostic, Swiss Tropical and Public Health Institute, Basel, Switzerland; 9 Institute for Social and Preventive Medicine, University of Zurich, Zurich, Switzerland; The George Washington University, United States of America

## Abstract

**Background:**

Infections with *Strongyloides stercoralis* and other helminths represent important, yet often neglected issues in developing countries. Indeed, strongyloidiasis can be fatal, but only a few studies provide information regarding its health relevance in Africa. Moreover, clinical data on symptomatology and typical recognition patterns mainly originate from Western travel clinics.

**Methodology:**

A cross-sectional epidemiological survey was carried out in a rural part of south-central Côte d'Ivoire. Stool samples from 292 randomly selected individuals were examined for intestinal helminths, using a suite of diagnostic techniques (i.e., Kato-Katz, Baermann funnel, and Koga agar plate). Participants were interviewed with a pre-tested questionnaire and clinically examined. Multivariate logistic regression analysis was done to relate perceived morbidity and clinical findings to helminth infection status.

**Principal Findings:**

The prevalence of hookworm and *S. stercoralis* was 51.0% and 12.7%, respectively. Both infections were strongly associated with each other (adjusted odds ratio, 6.73; *P*<0.001) and higher prevalences were observed with age. *S. stercoralis*-infected individuals expressed self-reported morbidity considerably more often than those with hookworm infection. Clinical examination identified high prevalences of various pathologies and detected tendencies to worse health conditions in helminth-infected subjects.

**Conclusions/Significance:**

The use of multiple diagnostic tools showed that *S. stercoralis* and hookworm are co-endemic in rural Côte d'Ivoire and that each infection causes clinical symptoms and sequelae. Our findings are important for (re-)estimating the burden of helminth infections, and highlight the need for integrating epidemiological surveys, rigorous diagnostic approaches, and clinical assessments in the developing world.

## Author Summary

Infections with parasitic worms such as hookworm and threadworm (*Strongyloides stercoralis*) are widespread throughout the developing world. However, the symptoms caused by parasitic worms are unspecific and little is known about clinical presentations in endemic countries, and hence doctors' awareness of these diseases is usually low. Many infections therefore remain undetected and untreated over long time periods. As a consequence, parasitic worms can impair the well-being of infected individuals and cause harmful, sometimes even fatal health outcomes. To improve the knowledge about clinical signs and symptoms caused by parasitic worms, we administered a questionnaire to 292 children and adults in rural Côte d'Ivoire, examined them clinically, and looked at their stool for parasitic worm infections. We found that people with parasitic worms reported some symptoms, especially disorders of the gastrointestinal tract, more often than those without worms. Our clinical examination showed a trend toward worse health conditions in worm-infected people, particularly in those with *S. stercoralis*. Our results are important to improve patient management and control measures, and to better estimate the true health impact of parasitic worm infections.

## Introduction

Infections with the nematodes *Strongyloides stercoralis* and hookworm (*Ancylostoma duodenale* and *Necator americanus*) are widespread in tropical and subtropical areas, accounting for a remarkable, yet underappreciated burden [1]. For example, hookworm infection is a leading cause of anemia [2], whereas *S. stercoralis* is able to maintain itself for decades within its host and may cause a lethal hyperinfection syndrome among immunosuppressed patients [3], [4]. Both infections belong to the heterogeneous group of the neglected tropical diseases (NTDs), which are notoriously under-researched and control efforts not adequately funded. Lately, more attention has been addressed to the NTDs [5], [6], which is justified by their cumulative global burden (>50 million disability-adjusted life years (DALYs) lost annually) [7]. The ‘true’ burden of the NTDs might even be higher [8], but considerable uncertainty remains. For example, estimates regarding the global burden due to hookworm infection range between 60,000 DALYs and 22.1 million DALYs, whereas no burden estimates are available for strongyloidiasis [9]. The lack of accurate diagnostic tools is an important consideration, which explains the often unsatisfactory epidemiological mapping of NTDs [10], [11]. With regard to strongyloidiasis, most information regarding signs, symptoms, and recognition patterns originate from examinations of returning travelers in Western travel clinics [12], and refugee populations in the North [13].

Surprisingly, biomedical research in endemic settings has mainly focused on chronic effects (e.g., stunting and nutritional deficiencies) of helminth infections, while little is known on acute impairment, despite the fact that clinical manifestations may profoundly differ between travelers and individuals from endemic areas. This has been shown for *S. stercoralis* [14], which may cause a severe and fatal hyperinfection syndrome, particularly in patients with HTLV-1 infection, on steroid medication and other immunosuppressive drugs, such as organ transplant recipients. While a higher suspicion index of strongyloidiasis is likely to improve early diagnosis and adequate treatment, epidemiological data are scarce in tropical countries [15]. It is currently estimated that between 30 and 100 million people are infected with *S. stercoralis* [1], but little is known about arising consequences in endemic areas. What symptoms does a *S. stercoralis* infection cause in tropical settings? When should a clinician suspect strongyloidiasis? Are individuals affected by hookworm or *S. stercoralis* prone to co-infections and co-morbidities? Answers to these questions will deepen our understanding of the true impact of nematode infections and result in improved patient management.

Here, we show a multi-pronged approach to elucidate clinical features of *S. stercoralis* and hookworm infections in a co-endemic area of Côte d'Ivoire. Our assessment is based on a cross-sectional epidemiological survey and in-depth laboratory investigations, complemented by a morbidity-centered questionnaire survey and standardized clinical examination.

## Methods

### Ethics Statement

The study protocol was approved by the institutional research commissions of the Swiss Tropical and Public Health Institute (Swiss TPH; Basel, Switzerland) and the Centre Suisse de Recherches Scientifiques en Côte d'Ivoire (CSRS; Abidjan, Côte d'Ivoire). Ethical approval was granted by the ethics committee of Basel (EKBB; reference no. 316/08), and the Ministry of Higher Education and Scientific Research in Côte d'Ivoire (reference no. 124/MESRS/DGRSIT/YKS/sac).

Political and health authorities were informed about the purpose and procedures of the study. Participants were informed about their involvement, including possible concerns and the right to withdraw at any moment without further obligations. Adult participants and the parents/legal guardians of participating children (aged<16 years) signed a written informed consent. At the end of the study, all individuals were invited to learn about their parasitological results and free treatment was offered to participants infected with helminths (i.e., single 400 mg dose of albendazole against hookworm and other soil-transmitted helminths, single 200 µg/kg dose of ivermectin against *S. stercoralis*, and single 40 mg/kg dose of praziquantel against *Schistosoma haematobium* and *Schistosoma mansoni*). Due to the high observed prevalence of soil-transmitted helminths, annual treatment with albendazole has been initiated in this study area in early 2010. Ivermectin is also being administered as part of the lymphatic filariasis control program.

### Study Area

The study was carried out in Léléblé and five surrounding hamlets (‘*campements*’) in May and June 2009. Léléblé is the second largest village that forms part of the recently established Taabo health demographic surveillance system (Taabo HDSS) in south-central Côte d'Ivoire, located approximately 160 km north of Abidjan. A census done at the end of 2008 revealed that the total population in Léléblé was 5,235 people. The tropical climate follows a seasonal pattern with a rainy season from May to October and a dry season from November to April. Taabo HDSS covers the area around Lake Taabo, a man-made reservoir inundated in the late 1970s [16]. Approximately 38,000 people live within the Taabo HDSS, consisting of a small district town (Taabo-Cité), 14 villages, including Léléblé, and over 100 small hamlets.

### Sample Size Calculation and Population Surveyed

In May 2009, a population-representative epidemiological survey was conducted in Taabo HDSS. Approximately 7% of the households were randomly selected; for Léléblé and surrounding hamlets this resulted in a sample size of 351 individuals. Allowing for a drop-out rate of 20–25%, we assumed that complete data records from at least 260 individuals would be available for appraisal of the population prevalence of *S. stercoralis* in Léléblé and surrounding hamlets with reasonable accuracy [17], [18].

### Field and Laboratory Procedures

Collection containers were distributed and study participants invited to submit a urine sample and a lemon-sized portion of their morning stool the following day. Samples were collected at a public spot between 08:00 and 12:00 hours and then transferred to a laboratory in Taabo-Cité, 28 km east of Léléblé.

Stool samples were examined for the presence of helminth eggs or larvae, using a suite of quality-controlled diagnostic methods: Kato-Katz [19], Baermann funnel (BM) [20], and Koga agar plate (KAP) [21]. Standard protocols of these techniques have been described elsewhere [22]. Additionally, for sufficiently large samples, ∼2 g of stool was put in 15 ml Falcon tubes filled with 10 ml of 5% formalin and, within 4 months, examined using the formalin-ether concentration technique (FECT), and the Flotac-400 dual technique. The comparison of these two methods for diagnosis of intestinal protozoon infections has been presented elsewhere [23].

From each stool sample, duplicate 41.7 mg Kato-Katz thick smears were prepared with slides read after a clearing time of 30–45 min. The number of helminth eggs was counted and recorded for each species separately. Infection intensity was derived by multiplying egg counts by a factor 24 and expressed as eggs per gram of stool (EPG). The exact number of observed hookworm and *S. stercoralis* larvae was recorded in the BM and the KAP tests.

Urine samples were analyzed for the presence of *S. haematobium* eggs. In brief, samples were vigorously shaken and 10 ml was filtered through a 13 µl nylon filter with a syringe. Filters were put on microscope slides, a drop of Lugol added, and *S. haematobium* eggs counted under a microscope by experienced laboratory technicians. For quality control, a random sample of ∼5% of all slides was re-examined by a senior technician.

### Questionnaire Survey

We searched the literature for common signs, symptoms, and complaints of hookworm and *S. stercoralis* infection. These symptoms were included in a questionnaire, which consisted of four parts: (i) demographic and anthropometric measures (e.g., sex, age, occupation, educational attainment, individual's height and weight); (ii) personal hygiene behavior (e.g., source of drinking water, frequency of contact with Lake Taabo, availability of toilets, regular use of soap, and wearing shoes); (iii) nine specific questions pertaining to past and recent medical history (e.g., chronic or infectious diseases such as asthma, diabetes, tuberculosis; hospitalization during the last 2 months; antimalarial and anthelmintic treatment in the last 2 months); and (iv) perceived state of health (recall period: 2 weeks), including questions concerning any disorders in the gastrointestinal tract (i.e., abdominal pain, abdominal distension, diarrhea, constipation, weight loss, abdominal cramping, nausea, vomiting, and blood in stool), the lungs (i.e., dyspnea, cough for more than 2 weeks, fatigue, uncommon expectoration), and the skin (i.e., cutaneous rash, migrating eruptions, pruritus). The questionnaire was pre-tested in a neighboring village. Study participants were interviewed by experienced field enumerators employed by Taabo HDSS.

### Clinical Examination

A standardized clinical examination was performed by the first two authors (SLB and BS). The examination comprised an evaluation of a participant's general habitus, conjunctival inspection, palpation of the abdomen and the thorax, cardiac and pulmonary auscultation, presence and grade of hepatomegaly and splenomegaly, and presence of jaundice. Moreover, the skin was carefully examined for any signs compatible with helminth infection (larva currens, larva migrans, rash, and pruritus).

### Statistical Analysis

Data were double-entered, cross-checked in Excel version 10.0 (edition 2002, Microsoft Corporation) and analyzed using STATA version 10.0 (StataCorp.; College Station, TX, USA). Stool or urine samples found positive for a specific helminth infection by any of the employed diagnostic techniques were considered as true-positive, leading to the prevalence results of each method. The combined results of the different techniques served as diagnostic ‘gold’ standard and were used as an estimate of the ‘true’ prevalence. Prevalence, sensitivity (proportion of true-positives identified as positive), and negative predictive value (NPV) were calculated, including 95% confidence intervals (CI) to quantify statistical uncertainty. Infection intensities were classified according to WHO thresholds [24] on the basis of the mean EPG. Distributional differences were assessed by Pearson's χ^2^ and Fisher's exact test, as appropriate. For statistical significance *P*<0.05 was used throughout.

Self-reported morbidity results from the questionnaire and findings from the clinical examination were analyzed for associations with helminth infections by univariate logistic regression and odds ratios (OR) were computed. Multivariate logistic regression modeling was used to estimate significant associations between *S. stercoralis* or hookworm infection and findings from the questionnaire survey and the clinical examination. Outcomes were defined as species-specific helminth infection. Possible explanatory variables were included in the final model if they (i) were biologically plausible, (ii) were present in at least 5% of the study participants, and (iii) showed an association (*P*<0.2) with infection status in a univariate logistic regression analysis. Adjusted ORs were calculated in order to reveal associations between a specific helminth infection and morbidity indicators.

## Results

### Study Cohort

Complete parasitological and clinical data were available from 292 out of 351 randomly selected individuals, owing to a compliance of 83.2%. There were more females (n = 155, 53.1%) than males (n = 137, 46.9%). The median age of our cohort was 13 years (mean, 20.1 years; range, 2 months to 75 years), thus constituting a representative sample of the village (46% of the population aged<15 years, according to the 2008 census).

### Helminth Infections and Comparison of Diagnostic Methods

Eight different helminth species were detected (Table 1). Hookworm was the predominant species; the overall prevalence was 51.0% and infection intensities were mainly light (<2,000 EPG, 95.1%). *S. stercoralis* larvae were found in fecal samples of 37 individuals (12.7%). *S. haematobium* and *Ascaris lumbricoides* showed prevalences of 8.5% and 5.1%, respectively. The remaining helminth species were found in less than 5% of the participants.

**Table 1:** Helminth infections among 292 individuals in Léléblé, south-central Côte d'Ivoire, in mid-2009.

	N	%	95% CI
Soil-transmitted helminth			
Hookworm	149	51.0	45.1–56.9
*Strongyloides stercoralis*	37	12.7	9.1–17.0
*Ascaris lumbricoides*	15	5.1	2.9–8.3
*Trichuris trichiura*	8	2.7	1.2–5.3
Other helminth species			
*Schistosoma haematobium* a	23	8.5	5.5–12.5
*Schistosoma mansoni*	14	4.8	2.7–7.9
*Hymenolepis nana* and *Taenia* spp.	9	3.1	1.4–5.8
*Enterobius vermicularis*	1	0.5	0.01–2.5

Helminth infections were determined by a suite of diagnostic techniques (i.e., duplicate Kato-Katz thick smears, single Koga agar plate (KAP) test, single Baermann (BM) test, Flotac-400 dual technique, and formalin-ether concentration technique (FECT)).

N, number of individuals found positive; CI, confidence interval.

a n = 270, based on standard 10 ml filtration of a single urine sample.

Table 2 shows the comparison of the BM and KAP methods for the diagnosis of *S. stercoralis*. While the BM technique identified 26 out of 37 infections, 17 infections were detected by the KAP test, and hence the BM technique showed a considerably higher sensitivity than KAP (70.3% *vs.* 46.0%). NPVs were similar for the two methods (95.9% *vs.* 92.7%). With regard to hookworm diagnosis, duplicate Kato-Katz thick smears showed a sensitivity of 69.1%, which was slightly higher than a single KAP test (61.8%). Compared to our diagnostic ‘gold’ standard, a combination of duplicate Kato-Katz thick smears and a single KAP test resulted in a prevalence of 46.2% (95% CI, 40.4–52.1%). While *S. stercoralis* infection rates did not differ significantly by sex, hookworm was more frequently observed in males than females (57.7% *vs.* 45.2%; χ^2^ = 4.55, *P* = 0.033; Table 3). The prevalence of both helminth infections increased with age. While only 5.2% of all children aged<5 years were diagnosed with *S. stercoralis* infection, 17.9% of all adolescents and young adults (aged 15–29 years), and every fifth adult and elderly (>45 years) were found positive for this helminth species. A similar relationship between age and infection prevalence was detected for hookworm; the prevalence rose from 36.8% in early childhood to 51.4% in school-aged children before reaching a plateau around 60% in adults. The infection prevalence in people working as farmers in agriculture or as merchants in trade was disproportionately higher than in individuals with other professions. A multivariate logistic regression analysis revealed a highly significant association between *S. stercoralis* and hookworm infection (adjusted OR, 6.73; 95% CI, 2.51–18.08, *P*<0.001).

**Table 2:** Diagnostic accuracy of different techniques for detection of hookworm and *Strongyloides stercoralis*.

Helminth species	Method			Diagnostic ‘gold’ standard
	Koga agar plate (KAP)	Kato-Katz (duplicate thick smears)	Kato-Katz+KAP	
Hookworm				
No. of detected infections	92	103	135	149
False-negative test results	57	46	14	0
Prevalence (95% CI)	31.5% (26.2–37.2%)	35.3% (29.8–41.1%)	46.2% (40.4–52.1%)	51.0% (45.1–56.9%)
Sensitivity (95% CI)	61.8% (53.4–69.6%)	69.1% (61.1–76.4%)	90.6% (84.7–94.8%)	100%
NPV	71.5%	75.7%	91.1%	100%

	Baermann funnel (BM)	Koga agar plate (KAP)		Diagnostic ‘gold’ standard
*Strongyloides stercoralis*				
No. of detected infections	26	17		37
False-negative test results	11	20		0
Prevalence (95% CI)	8.9% (5.9–12.8%)	5.8% (3.4–9.2%)		12.7% (9.1–17.0%)
Sensitivity (95% CI)	70.3% (54.8–85.7%)	46.0% (29.1–62.8%)		100%
NPV	95.9%	92.7%		100%

Study was carried out in Léléblé, south-central Côte d'Ivoire, in mid-2009. Final study cohort consisted of 292 individuals. Diagnosis of hookworm was done by duplicate Kato-Katz thick smear and the Koga agar plate (KAP) method, whereas S. stercoralis diagnosis was done by Baermann and KAP.

CI, confidence interval; NPV, negative predictive value.

**Table 3:** Prevalence of hookworm and *Strongyloides stercoralis* infection, stratified by sex, age, and occupation.

	Hookworm			*S. stercoralis*		
	N	%	*P* a	N	%	*P* a
Sex						
Male	79	57.7		19	13.9	
Female	70	45.1	0.033	18	11.6	0.563
Age (years)						
<5	21	36.8		3	5.2	
6–15	54	51.4		11	10.5	
16–25	19	67.9		5	17.9	
26–45	45	59.2		13	17.1	
>45	10	38.5	0.022	5	19.2	0.136
Occupation						
Farmer	81	61.4		23	17.4	
Trader	7	58.3		3	25.0	
Preschool, student	32	42.7		4	5.3	
Other	29	39.7	0.008	7	9.6	0.024

Data are presented for 292 individuals from Léléblé, south-central Côte d'Ivoire, who participated in a cross-sectional survey in mid-2009.

a Derived from Pearson's χ^2^ or Fisher's exact test, as appropriate.

### Risk Factors and Self-Reported Morbidity

Table 4 summarizes risk factors and health-related behaviors, in relation to an infection with either *S. stercoralis* or hookworm. Non-infected individuals stated more often to have any grade of school education (46%; *P* = 0.116), to have access to sanitation (36%; *P* = 0.108), and a recent history of anthelmintic treatment (9%; *P* = 0.008) compared to their infected counterparts. Among infected individuals, their stomach was more often considered as a major health problem (*S. stercoralis*-infected, 35%; hookworm-infected, 23%) than among individuals who had neither a hookworm nor a *S. stercoralis* infection (15%; *P* = 0.039). Different gastrointestinal symptoms such as severe abdominal pain, blood in stool, and diarrhea were more often reported among helminth-infected individuals.

**Table 4:** Health-related features and self-reported morbidity according to helminth infection status.

Feature/morbidity	Infection status						*P* a
	Hookworm(n = 149)		*S. stercoralis*(n = 37)		Free of hookworm and *S. stercoralis* infection(n = 136)		
	N	%	N	%	N	%	
Education, socio-economy, and recent treatment history							
Any school education	57	38.3	12	32.4	62	46.0	0.116
Access to sanitation	44	29.5	12	32.4	49	36.0	0.190
Anthelmintic treatment (last 2 months)	3	2.0	0	0	12	8.8	0.008
General health							
Poor state of health	37	24.8	11	29.7	19	14.0	0.013
Major health problem: abdominal pain	34	22.8	13	35.1	20	14.7	0.039
Gastrointestinal symptoms							
Severe abdominal pain	45	30.2	15	40.5	34	25.0	0.226
Diarrhea	35	23.5	7	18.9	18	13.2	0.022
Blood in stool	42	28.2	12	32.4	22	16.1	0.010
Pulmonary symptoms							
Cough	44	29.5	10	27.0	39	28.9	0.879
Dermatologic symptoms							
Skin eruption	18	12.1	6	16.2	16	11.8	0.952
Cutaneous pruritus	26	17.5	6	16.2	22	16.2	0.910
Any of these symptoms	123	82.6	29	78.4	100	74.0	

Data were obtained from a cross-sectional survey carried out in Léléblé, south-central Côte d'Ivoire, in mid-2009.

a Cross-tabulation, *P* according to χ^2^ test.

Multivariate logistic regression analysis revealed that some risk factors and symptoms were associated with *S. stercoralis*, hookworm, or both infections concurrently (Table 5). Individuals complaining about frequent stomach ache (adjusted OR, 2.35; 95% CI, 0.98–5.62, *P* = 0.056) were more likely to be infected with *S. stercoralis*, while self-reported diarrhea (adjusted OR, 1.89; 95% CI, 0.98–3.65, *P* = 0.057) and “working as a farmer in agriculture” (adjusted OR, 2.62; 95% CI, 1.27–5.40, *P* = 0.009) were risk factors for a hookworm infection.

**Table 5:** Significant associations between hookworm or *S. stercoralis* infection and health-related behaviors and perceived morbidity indicators.

Helminth species	Feature	N	OR	95% CI	*P*
Hookworm	Occupation: farmer	81	2.62	1.27–5.40	0.009
	Recent anthelmintic treatment	3	0.26	0.07–1.03	0.056
	Diarrhea	35	1.89	0.98–3.65	0.057
*Strongyloides stercoralis*	Use of community tap water	35	6.18	1.36–28.16	0.019
	Major health complaint: stomach ache	13	2.35	0.98–5.62	0.056

Data were obtained from a cross-sectional survey with 292 participating individuals in Léléblé, south-central Côte d'Ivoire, in mid-2009. Shown are the results from multivariate logistic regression analyses, controlling for age, sex, and residency.

CI, confidence interval; OR, odds ratio.

### Clinical Examination

Hepatomegaly and splenomegaly were frequently observed in the study population with prevalences of 31.8% and 31.2%, respectively. Abnormal findings on pulmonary auscultation (i.e., wheezing, rhonchi, crackles and rales, bronchial breathing over the chest) were noted in one out of five participants (20.5%).

**Figure 1:**
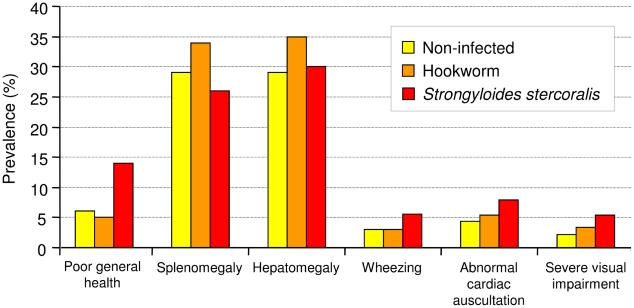
Clinical characterization of 292 individuals in Léléblé, south-central Côte d'Ivoire, in mid-2009. Shown on this figure are the prevalences of clinical signs and symptoms among 292 study participants from Léléblé who had complete data records, according to their infection status with hookworm and *Strongyloides stercoralis*.

We only found numeric, non-significant differences between *S. stercoralis*-infected, hookworm-infected, and participants with neither of these infections (Figure 1). A general poor state of health characterized by inadequate personal hygiene and squalidness was more frequent in *S. stercoralis*-infected individuals (13.5% *vs.* 4.7%), as well as the uncommon sign “wheezing on pulmonary auscultation” (5.4% *vs.* 2.7%). However, a combination of such signs was seldom present, thus hampering an exact differentiation of helminth infections by a combination of specific signs. More than half (54.1%) of all *S. stercoralis*-infected individuals were found to suffer from either pulmonary wheezing, abdominal pain (as determined by the questionnaire survey or found on examination), or a general malaise, while one of these three signs was present in only 26.9% of their hookworm-infected counterparts. Anthropometrics revealed no statistically significant differences between helminth-infected and non-infected individuals (data not shown).

## Discussion

The study reported here from a rural part of south-central Côte d'Ivoire confirms that hookworm and *S. stercoralis* are co-endemic [25]. While every second participant was infected with hookworm, *S. stercoralis* infections were found in 12.7% of our cohort. Interestingly, *S. stercoralis* was exclusively found in the main study village (Léléblé), but not in the surrounding hamlets, indicating that this nematode species requires distinct environmental and biologic characteristics to cause human infection. Moreover, we found that both hookworm and *S. stercoralis* disproportionately affect the poorest population segments, i.e., those with no or limited education, lack of personal hygiene, and the worst self-reported and clinically-examined health conditions. While the prevalence of hookworm increases with age, other helminths mainly affect school-aged children [2]. In the present work, the *S. stercoralis* age-prevalence curve showed an almost parallel shape to the hookworm age-prevalence curve, reaching an infection rate of approximately 20% in older adults.

The highly significant association between hookworm and *S. stercoralis* (OR>6), and the series of self-reported morbidity markers associated with these two helminth species are particularly noteworthy. Interestingly, the perceived health impact of strongyloidiasis appeared to be greater than morbidity due to a hookworm infection, especially regarding gastrointestinal symptoms, skin rash, and abnormal wheezing on pulmonary auscultation. These findings are coherent with different disease stages of strongyloidiasis, thus indicating an entitative and reproducibly measurable morbidity in infected subjects who live in endemic areas. This observation opposes the widely expressed opinion that most *S. stercoralis*-infected individuals residing in tropical endemic areas remain asymptomatic [3]. The assessment of acute, impaired well-being is a relevant consideration for the development of accurate disease burden estimates for strongyloidiasis and hookworm disease, particularly in view of the often contradictory data on the long-term effects due to soil-transmitted helminthiasis, e.g., cognitive performance and physical development of infected school-aged children [26]–[29]. Factual data derived from clinical examinations in endemic areas may be more appropriate than questionnaire surveys investigating self-reported morbidity, because many complaints, which are commonly imputed to helminth infections, are also frequently caused by other infective agents and/or nutritional deficiencies. Moreover, medical examinations in zones of rural poverty may allow identification and differentiation of certain helminth infections by a distinct cluster of clinical signs, thus superseding the need for detailed laboratory investigations, which are seldom available in resource-constrained areas.

Our study suffers from several limitations that are offered for consideration. First, the prevalence of hookworm and *S. stercoralis* infections is likely to be underestimated due to (i) the low sensitivity of stool microscopy [10], [22], and (ii) the examination of only one stool sample per person, as the output of helminth eggs and larvae in fecal material shows considerable day-to-day variation [22], [30]. However, we used a suite of diagnostic techniques to enhance sensitivity, e.g., KAP test and BM technique for the diagnosis of *S. stercoralis*. The BM funnel, which is considered the method of choice for *S. stercoralis* diagnosis when analyzing stool samples [31], was indeed more sensitive than the KAP test, confirming recent studies carried out in the People's Republic of China [32], Zanzibar [22], [33], and Uganda [34]. However, accurate diagnosis remains a critical issue, even when techniques other than stool microscopy are employed [35]. The usefulness of serology in endemic areas has been discussed controversially [36], and a recently developed polymerase chain reaction (PCR) assay [37], which may become a new diagnostic ‘gold’ standard, needs to be validated in different epidemiological settings prior to larger scale use. Molecular techniques still have a long way to go until they might become viable alternatives for diagnosis of NTDs that are intimately connected to poverty. Second, a larger sample size may have revealed a more distinct clinical recognition pattern of the helminth infections investigated here, particularly for *S. stercoralis*, which we expected to be even more prevalent in the study region, based on preliminary results obtained from five villages in the Taabo HDSS study area [25].

In conclusion, *S. stercoralis* and hookworm infections represent important health threats in tropical regions and both parasites cause morbid sequelae. While especially strongyloidiasis is increasingly recognized as an important cause of morbidity and persistent mortality in some parts of the world [4], its diagnosis remains a challenge and clinical features in resource-constrained settings are still neglected [35], [38]. Our study revealed the difficulty to properly assess helminth infections and proposes clinical in-depth studies to factually substantiate the estimated helminth disease burden in order to obtain a more accurate picture of their relevance in Africa. In view of changing health care patterns in tropical countries, including broader access to steroid treatment, an increasing number of the potentially fatal hyperinfection syndrome has to be expected in Africa and elsewhere. Hence, there is a need to raise physicians' awareness of strongyloidiasis to finally improve the patient management and outcome of this important, but widely underrecognized infection.

## Supporting Information

Alternative Language Abstract S1 Diagnostik, klinische Charakteristika und Morbidität von Infektionen mit *Strongyloides stercoralis* und Hakenwürmern in einem co-endemischen Gebiet – Translation of abstract into German by Sören Becker.
(DOC)

Alternative Language Abstract S2 Diagnostic, Caractéristiques Cliniques et Morbidité due à des Infestations par *Strongyloides stercoralis* et les ankylostomes dans un Foyer de Co-endémicité – Translation of abstract into French by Kigbafori D. Silué.
(DOC)

## References

[1] Bethony J, Brooker S, Albonico M, Geiger SM, Loukas A (2006). Soil-transmitted helminth infections: ascariasis, trichuriasis, and hookworm.. Lancet.

[2] Hotez PJ, Brooker S, Bethony JM, Bottazzi ME, Loukas A (2004). Hookworm infection.. N Engl J Med.

[3] Segarra-Newnham M (2007). Manifestations, diagnosis, and treatment of *Strongyloides stercoralis* infection.. Ann Pharmacother.

[4] Croker C, Reporter R, Redelings M, Mascola L (2010). Strongyloidiasis-related deaths in the United States, 1991–2006.. Am J Trop Med Hyg.

[5] Feasey N, Wansbrough-Jones M, Mabey DC, Solomon AW (2010). Neglected tropical diseases.. Br Med Bull.

[6] Vanderelst D, Speybroeck N (2010). Quantifying the lack of scientific interest in neglected tropical diseases.. PLoS Negl Trop Dis.

[7] Hotez PJ, Molyneux DH, Fenwick A, Ottesen E, Ehrlich Sachs S (2006). Incorporating a rapid-impact package for neglected tropical diseases with programs for HIV/AIDS, tuberculosis, and malaria.. PLoS Med.

[8] King CH, Bertino AM (2008). Asymmetries of poverty: why global burden of disease valuations underestimate the burden of neglected tropical diseases.. PLoS Negl Trop Dis.

[9] Utzinger J, Raso G, Brooker S, de Savigny D, Tanner M (2009). Schistosomiasis and neglected tropical diseases: towards integrated and sustainable control and a word of caution.. Parasitology.

[10] Bergquist R, Johansen MV, Utzinger J (2009). Diagnostic dilemmas in helminthology: what tools to use and when?. Trends Parasitol.

[11] Johansen MV, Sithithaworn P, Bergquist R, Utzinger J (2010). Towards improved diagnosis of zoonotic trematode infections in Southeast Asia.. Adv Parasitol.

[12] Nuesch R, Zimmerli L, Stockli R, Gyr N, Hatz CFR (2005). Imported strongyloidosis: a longitudinal analysis of 31 cases.. J Travel Med.

[13] Posey DL, Blackburn BG, Weinberg M, Flagg EW, Ortega L (2007). High prevalence and presumptive treatment of schistosomiasis and strongyloidiasis among African refugees.. Clin Infect Dis.

[14] Sudarshi S, Stumpfle R, Armstrong M, Ellman T, Parton S (2003). Clinical presentation and diagnostic sensitivity of laboratory tests for *Strongyloides stercoralis* in travellers compared with immigrants in a non-endemic country.. Trop Med Int Health.

[15] Knopp S, Mohammed KA, Rollinson D, Stothard JR, Khamis IS (2009). Changing patterns of soil-transmitted helminthiases in Zanzibar in the context of national helminth control programs.. Am J Trop Med Hyg.

[16] N'Goran EK, Diabate S, Utzinger J, Sellin B (1997). Changes in human schistosomiasis levels after the construction of two large hydroelectric dams in central Côte d'Ivoire.. Bull World Health Organ.

[17] Naing L, Winn T, Rusli BN (2006). Practical issues in calculating the sample size for prevalence studies.. Arch Orofac Sci.

[18] Daniel WW (1999). Biostatistics: A Foundation for Analysis in the Health Sciences. 7th edition.

[19] Katz N, Chaves A, Pellegrino J (1972). A simple device for quantitative stool thick-smear technique in schistosomiasis mansoni.. Rev Inst Med Trop São Paulo.

[20] García LS, Bruckner DA (2001). Diagnostic Medical Parasitology.

[21] Koga K, Kasuya S, Khamboonruang C, Sukhavat K, Ieda M (1991). A modified agar plate method for detection of *Strongyloides stercoralis*.. Am J Trop Med Hyg.

[22] Knopp S, Mgeni AF, Khamis IS, Steinmann P, Stothard JR (2008). Diagnosis of soil-transmitted helminths in the era of preventive chemotherapy: effect of multiple stool sampling and use of different diagnostic techniques.. PLoS Negl Trop Dis.

[23] Becker SL, Lohourignon LK, Speich B, Rinaldi L, Knopp S (2011). Comparison of the Flotac-400 dual technique and the formalin-ether concentration technique for diagnosis of human intestinal protozoon infection.. J Clin Microbiol.

[24] WHO (2002). Prevention and control of schistosomiasis and soil-transmitted helminthiasis: report of a WHO expert committee.. WHO Tech Rep Ser.

[25] Glinz D, N'Guessan NA, Utzinger J, N'Goran EK (2010). High prevalence of *Strongyloides stercoralis* among school children in rural Côte d'Ivoire.. J Parasitol.

[26] Dickson R, Awasthi S, Demellweek C, Williamson P (2007). WITHDRAWN: Anthelmintic drugs for treating worms in children: effects on growth and cognitive performance.. Cochrane Database Syst Rev.

[27] Ziegelbauer K, Steinmann P, Zhou H, Du ZW, Jiang JY (2010). Self-rated quality of life and school performance in relation to helminth infections: case study from Yunnan, People's Republic of China.. Parasit Vectors.

[28] Müller I, Coulibaly JT, Fürst T, Knopp S, Hattendorf J (2011). Effect of schistosomiasis and soil-transmitted helminth infections on physical fitness of school children in Côte d'Ivoire.. PLoS Negl Trop Dis.

[29] Fürst T, Müller I, Coulibaly JT, Yao AK, Utzinger J (2011). Questionnaire-based approach to assess schoolchildren's physical fitness and its potential role in exploring the putative impact of helminth and *Plasmodium* spp. infections in Côte d'Ivoire.. Parasit Vectors.

[30] Utzinger J, Booth M, N'Goran EK, Müller I, Tanner M (2001). Relative contribution of day-to-day and intra-specimen variation in faecal egg counts of *Schistosoma mansoni* before and after treatment with praziquantel.. Parasitology.

[31] WGO (2004). WGO Practice Guideline Management of Strongyloidiasis.. http://www.worldgastroenterology.org/assets/downloads/en/pdf/guidelines/15_management_strongyloidiasis_en.pdf.

[32] Steinmann P, Zhou XN, Du ZW, Jiang JY, Wang LB (2007). Occurrence of *Strongyloides stercoralis* in Yunnan province, China, and comparison of diagnostic methods.. PLoS Negl Trop Dis.

[33] Knopp S, Mohammed KA, Khamis IS, Mgeni AF, Stothard JR (2008). Spatial distribution of soil-transmitted helminths, including *Strongyloides stercoralis*, among children in Zanzibar.. Geospat Health.

[34] Stothard JR, Pleasant J, Oguttu D, Adriko M, Galimaka R (2008). *Strongyloides stercoralis*: a field-based survey of mothers and their preschool children using ELISA, Baermann and Koga plate methods reveals low endemicity in western Uganda.. J Helminthol.

[35] Montes M, Sawhney C, Barros N (2010). *Strongyloides stercoralis*: there but not seen.. Curr Opin Infect Dis.

[36] Siddiqui AA, Berk SL (2001). Diagnosis of *Strongyloides stercoralis* infection.. Clin Infect Dis.

[37] Verweij JJ, Canales M, Polman K, Ziem J, Brienen EA (2009). Molecular diagnosis of *Strongyloides stercoralis* in faecal samples using real-time PCR.. Trans R Soc Trop Med Hyg.

[38] Olsen A, van Lieshout L, Marti H, Polderman T, Polman K (2009). Strongyloidiasis – the most neglected of the neglected tropical diseases?. Trans R Soc Trop Med Hyg.

