# Supplementary material for: Diagnosis, Clinical Features, and Self-Reported Morbidity of Strongyloides stercoralis and Hookworm Infection in a Co-Endemic Setting
Source: PLoS Negl Trop Dis. 2011 Aug 23;5(8):e1292. doi: 10.1371/journal.pntd.0001292 (PMC3160297; doi:10.1371/journal.pntd.0001292)
Supplement: Alternative Language Abstract S1 — Diagnostik, klinische Charakteristika und Morbidität von Infektionen mit Strongyloides stercoralis und Hakenwürmern in einem co-endemischen Gebiet – Translation of abstract into German by Sören Becker. (DOC) [file pntd.0001292.s001.doc]

**Diagnostik, klinische Charakteristika und Morbidität von Infektionen mit** *Strongyloides stercoralis* **und Hakenwürmern in einem co-endemischen Gebiet**

**Zusammenfassung**

***Hintergrund:*** Infektionen mit *Strongyloides stercoralis* und anderen parasitären Würmern stellen bedeutende, jedoch oft vernachlässigte Gesundheitsprobleme in Entwicklungsländern dar. Obwohl die Strongyloidiasis zu tödlichen Verläufen führen kann, haben bisher nur wenige Studien die medizinische Bedeutung dieser Erkrankung in Afrika untersucht. Darüber hinaus stammen die meisten Informationen bezüglich typischer Symptome und klinischer Charakteristika von reisemedizinischen Zentren ausserhalb tropischer Endemiegebiete.

***Methodologie:*** Wir führten eine epidemiologische Querschnittsstudie in einer ländlichen Region im Süden der Côte d’Ivoire durch, in deren Rahmen Stuhlproben von 292 zufällig ausgewählten Individuen mittels verschiedener diagnostischer Techniken (Kato-Katz, Baermann Test, Koga Agarplatte) auf Infektionen mit parasitären Würmern untersucht wurden. Ausserdem wurden Studienteilnehmer mit einem standardisierten Fragebogen interviewt und klinisch-körperlich untersucht, um eventuell bestehende Pathologien zu dokumentieren. Mögliche Assoziationen zwischen Wurminfektionen und gemessener Morbidität wurden mittels multivariabler logistischer Regressionsanalysen untersucht.

***Wichtigste Ergebnisse:*** Die Infektionsprävalenz betrug 51.0% für Hakenwürmer und 12.7% für *S. stercoralis*. Beide Infektionen waren sehr deutlich miteinander assoziiert (adjustierte Odds Ratio: 6.73, *P*<0.001) und zeigten die höchsten Infektionsraten bei älteren Studienteilnehmern und -teilnehmerinnen. Individuen mit einer *S. stercoralis*-Infektion berichteten häufiger über körperliche Symptome als die mit einer Hakenwurm-Infektion. Ausserdem deckte die körperliche Untersuchung hohe Prävalenzen verschiedener Pathologien auf und dokumentierte eine Tendenz zu schlechteren Gesundheitszuständen in Individuen mit Wurminfektionen.

***Schlussfolgerungen/Bedeutung:*** Die Kombination verschiedener diagnostischer Techniken ergab, dass *S. stercoralis* und Hakenwürmer co-endemisch in diesem ländlichen Gebiet der Côte d’Ivoire sind, und dass beide Infektionen zu Symptomen mit klinisch messbaren Gesundheitseinschränkungen führen. Diese Erkenntnisse sind für eine realistische Einschätzung der weltweiten Krankheitsbürde durch Wurminfektionen bedeutsam. Des Weiteren unterstreicht unsere Studie die Wichtigkeit eines integrierten Ansatzes zur Erfassung der Gesundheitsauswirkungen parasitärer Erkrankungen in Entwicklungsländern, wobei epidemiologische Studien mit sensitiven diagnostischen Techniken und klinischen Datenerhebungen kombiniert werden sollten.

***Übersetzung:*** Sören L. Becker
