# Supplementary material for: Diagnosis, Clinical Features, and Self-Reported Morbidity of Strongyloides stercoralis and Hookworm Infection in a Co-Endemic Setting
Source: PLoS Negl Trop Dis. 2011 Aug 23;5(8):e1292. doi: 10.1371/journal.pntd.0001292 (PMC3160297; doi:10.1371/journal.pntd.0001292)
Supplement: Alternative Language Abstract S2 — Diagnostic, Caractéristiques Cliniques et Morbidité due à des Infestations par Strongyloides stercoralis et les ankylostomes dans un Foyer de Co-endémicité – Translation of abstract into French by Kigbafori D. Silué. (DOC) [file pntd.0001292.s002.doc]

**Diagnostic, Caractéristiques Cliniques et Morbidité due à des Infestations par *Strongyloides stercoralis* et les ankylostomes dans un Foyer de Co-endémicité**

**Résumé**

***Contexte :*** Les infestations par *Strongyloides stercoralis* et autres helminthes sont un problème de santé publique, mais hélas bien souvent négligées dans les pays en voie de développement. En effet, la strongyloïdiase peut être mortelle, mais il n’y a que peu d'études qui renseignent sur son impact en santé publique en Afrique. Cependant, les données cliniques sur la symptomatologie et les aspects typiques d’identification proviennent principalement de cliniques occidentales de voyage.

***Méthodologie :*** Une enquête épidémiologique transversale a été conduite en milieu rural au centre-sud de la Côte d'Ivoire. Les échantillons de selles de 292 personnes choisies de façon aléatoire ont été examinés pour la recherche d’helminthes intestinaux à partir d’un ensemble de techniques de diagnostic (Kato-Katz, technique de Baermann et technique de Koga). Les participants ont été interviewés à l’aide d’un questionnaire pré-testé suivi d’un examen clinique. La régression logistique multivariée a été appliquée afin d’établir le lien entre le statut d’infestation par les helminthes, la morbidité perçue et les résultats cliniques.

***Principaux Résultats :*** La prévalence d’ankylostomes et de *S. stercoralis* a été de 51,0% et 12,7%, respectivement. Les deux infestations ont été fortement associées entre elles (odds ratio ajusté = 6,73; P <0,001) et les plus fortes prévalences ont été observées avec l'âge. Les participants infestés par *S. stercoralis* ont rapporté la morbidité nettement plus souvent que ceux infestés par les ankylostomes. L'examen clinique a permis d’identifier des prévalences élevées de pathologies diverses et de détecter des tendances à de mauvaises conditions de santé chez les personnes infestées par les helminthes.

***Conclusion******:***L'utilisation de plusieurs outils de diagnostic a montré que *S. stercoralis* et les ankylostomes sont co-endémiques en milieu rural en Côte d'Ivoire et que chaque infestation cause des symptômes cliniques et des séquelles. Nos résultats sont importants pour une (re-) estimation du fardeau d’infestations majeures par les helminthes et soulignent la nécessité d'étude épidémiologique intégrées, des approches rigoureuses de diagnostic et d’évaluations cliniques dans les pays en voie de développement.

***Traduction :*** Kigbafori D. Silué
